# An Electronic Clinical Decision Support System for the Management of Low Back Pain in Community Pharmacy: Development and Mixed Methods Feasibility Study

**DOI:** 10.2196/17203

**Published:** 2020-05-11

**Authors:** Aron Simon Downie, Mark Hancock, Christina Abdel Shaheed, Andrew J McLachlan, Ahmet Baki Kocaballi, Christopher M Williams, Zoe A Michaleff, Chris G Maher

**Affiliations:** 1 Institute for Musculoskeletal Health Sydney School of Public Health, Faculty of Medicine and Health The University of Sydney Camperdown Australia; 2 Faculty of Science and Engineering Macquarie University Macquarie Park Australia; 3 Faculty of Medicine, Health and Human Sciences Macquarie University Macquarie Park Australia; 4 Sydney Pharmacy School, Faculty of Medicine and Health University of Sydney Sydney Australia; 5 Centre for Health Informatics, Australian Institute of Health Innovation Faculty of Medicine and Health Sciences Macquarie University Macquarie Park Australia; 6 Faculty of Engineering and Information Technology University of Technology Sydney Sydney Australia; 7 Hunter New England Population Health Hunter New England Local Health District Newcastle Australia; 8 Institute for Evidence-Based Healthcare, Faculty of Health Sciences and Medicine Bond University Gold Coast Australia

**Keywords:** low back pain, community pharmacy, decision support systems, clinical

## Abstract

**Background:**

People with low back pain (LBP) in the community often do not receive evidence-based advice and management. Community pharmacists can play an important role in supporting people with LBP as pharmacists are easily accessible to provide first-line care. However, previous research suggests that pharmacists may not consistently deliver advice that is concordant with guideline recommendations and may demonstrate difficulty determining which patients require prompt medical review. A clinical decision support system (CDSS) may enhance first-line care of LBP, but none exists to support the community pharmacist–client consultation.

**Objective:**

This study aimed to develop a CDSS to guide first-line care of LBP in the community pharmacy setting and to evaluate the pharmacist-reported usability and acceptance of the prototype system.

**Methods:**

A cross-platform Web app for the Apple iPad was developed in conjunction with academic and clinical experts using an iterative user-centered design process during interface design, clinical reasoning, program development, and evaluation. The CDSS was evaluated via one-to-one user-testing with 5 community pharmacists (5 case vignettes each). Data were collected via video recording, screen capture, survey instrument (system usability scale), and direct observation.

**Results:**

Pharmacists’ agreement with CDSS-generated self-care recommendations was 90% (18/20), with medicines recommendations was 100% (25/25), and with referral advice was 88% (22/25; total 70 recommendations). Pharmacists expressed uncertainty when screening for serious pathology in 40% (10/25) of cases. Pharmacists requested more direction from the CDSS in relation to automated prompts for user input and page navigation. Overall system usability was rated as excellent (mean score 92/100, SD 6.5; 90th percentile compared with similar systems), with acceptance rated as good to excellent.

**Conclusions:**

A novel CDSS (high-fidelity prototype) to enhance pharmacist care of LBP was developed, underpinned by clinical practice guidelines and informed by a multidisciplinary team of experts. User-testing revealed a high level of usability and acceptance of the prototype system, with suggestions to improve interface prompts and information delivery. The small study sample limits the generalizability of the findings but offers important insights to inform the next stage of system development.

## Introduction

### Background

Low back pain (LBP) is a major cause of disability worldwide [1], with almost 1 in 5 people reporting LBP at any one time [2]. People with LBP typically consult general practice, allied health, or community pharmacy for advice and management [3,4]. The role of community pharmacists has evolved from dispensing medication and providing medication advice, to include screening and management for a range of health conditions such as minor ailments and chronic health conditions [5-10]. In alignment with this expanding service model, there is interest for community pharmacy to play a greater role in the early management of back pain [11-13]. There are also potential economic benefits for using community pharmacy as an access point for a range of services, with lower patient and health system costs compared with other primary care models [13-15].

### Evidence-Practice Gaps in Management of Low Back Pain

Current clinical practice guidelines for the management of LBP recommend first-line care that includes reassurance, advice to stay active and avoid bed rest, and discouraging diagnostic imaging such as plain radiographs unless serious pathology is suspected [3,16]. Despite these guideline recommendations, a substantial gap between evidence and practice still exists [17]. For example, Abdel Shaheed et al [18] reported that community pharmacists and their staff were able to deliver adequate advice on medication use for LBP, but their ability to provide advice on nonpharmacological management such as staying active, avoiding bed rest, and discouraging imaging was inconsistently delivered. The ability to identify presentations that required prompt medical review was also limited for some community pharmacists.

### Support for the Community Pharmacist

Clinical decision support systems (CDSSs) are targeted electronic systems that link evidence-based recommendations with the clinical presentation of the individual to improve clinical decision making and support patient engagement with health decisions [19-24]. Recently, CDSSs have been implemented for management of noncancer pain in the primary care setting [25-29], but these do not transfer to the pharmacy setting because of differences in professional training and consultation environment. Pharmacists already have access to CDSSs (eg, management of infection and deprescribing) [30,31], but none exist to support the community pharmacist–client consultation for LBP. Therefore, a CDSS for the early management of LBP in community pharmacy is warranted [32].

The main objective of this study was to develop a CDSS for pharmacists to guide first-line care of LBP in the community pharmacy setting using a mobile data collection system (Apple iPad, Apple Inc). We also sought to evaluate the pharmacist-reported usability and acceptance of the high-fidelity prototype to inform the next stage of CDSS development.

## Methods

### Overview

This study describes the iterative development of a CDSS for the management of pharmacy clients with LBP in community pharmacy. The CDSS was developed by a multidisciplinary team that included two pharmacy academics, a human-computer interaction expert, and four content experts in LBP [33]. Team members were consulted during each stage of development. The CDSS (high-fidelity prototype) was evaluated via a small-scale usability study [34]. The study was approved by University of Sydney Human Research Ethics Committee (2017/027).

### User-Centered Design Framework

**Figure 1:**
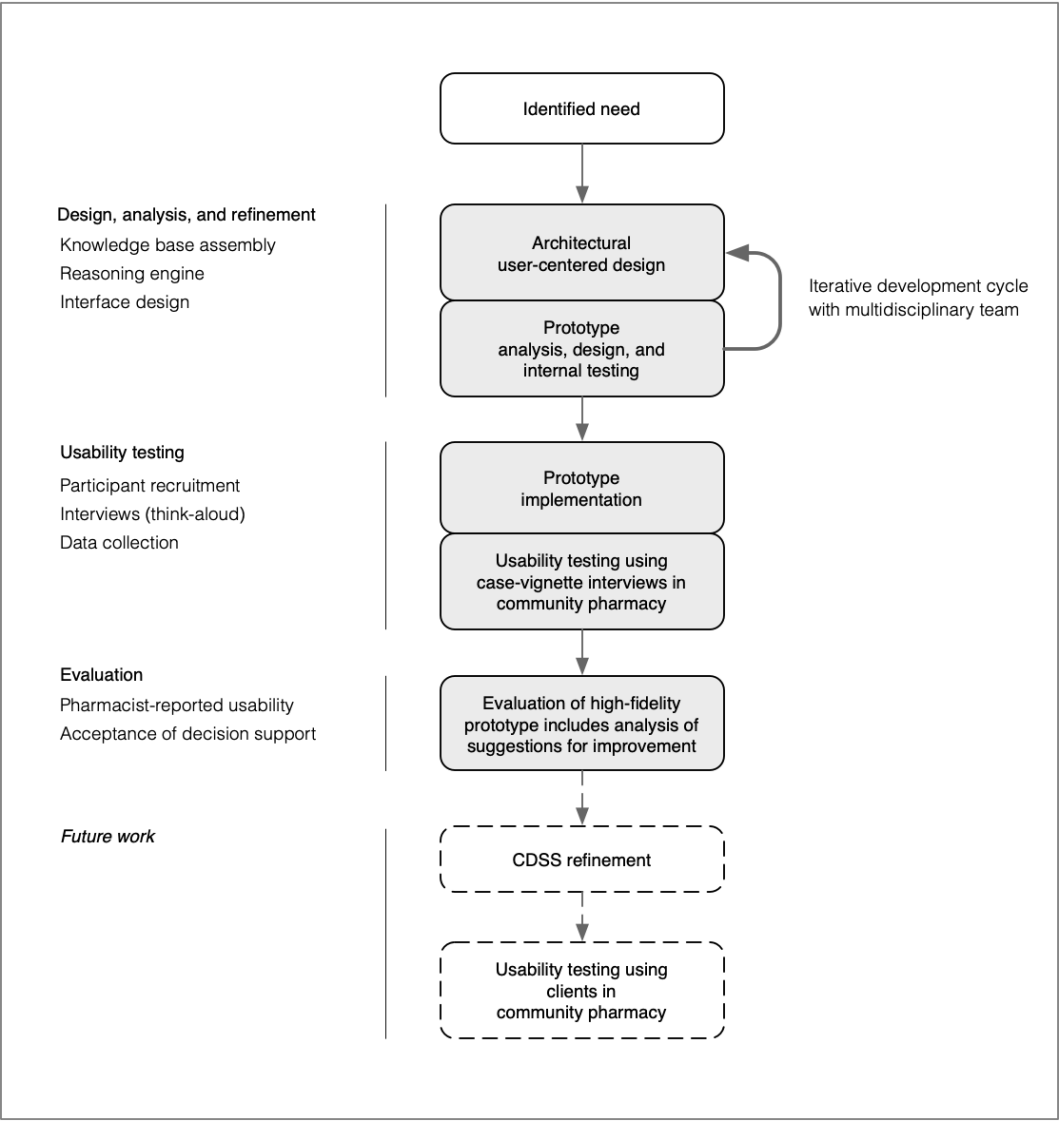
Clinical decision support system development based on prototyping and iterative testing (modified from Kushniruk et al). This study is represented by shading. CDSS: clinical decision support system.

Development of the CDSS was underpinned by the framework for user-centered design and evaluation of prototypes for clinical information systems (Figure 1) [35]. The framework describes the evolution of a CDSS based around low-cost usability testing methods before future evaluation with real clients in a clinical practice setting. During initial design of the CDSS, input was sought from a range of people involved with community pharmacy, including two pharmacy academic/educators, a community pharmacist, and an industry representative. This approach sought to uncover pharmacist training and procedural constraints that may impact pharmacist decision making for LBP [36], given that a pathway for the contemporary management of LBP specific to the community pharmacy setting does not exist.

### Architectural Design, Analysis, and Refinement

Design goals were informed by Bates et al [37], Khorasani et al [38], and Zikos et al [39] who described features of a decision support system necessary to facilitate integration into clinical practice. The design goals of this CDSS were to (1) support pharmacists to offer simple, clear evidence-based advice to the pharmacy client who presents with LBP; (2) integrate with the pharmacist workflow (eg, consideration of medicines during decision making); (3) maximize time efficiency; and (4) provide a personalized report of recommendations for the pharmacy client.

**Figure 2:**
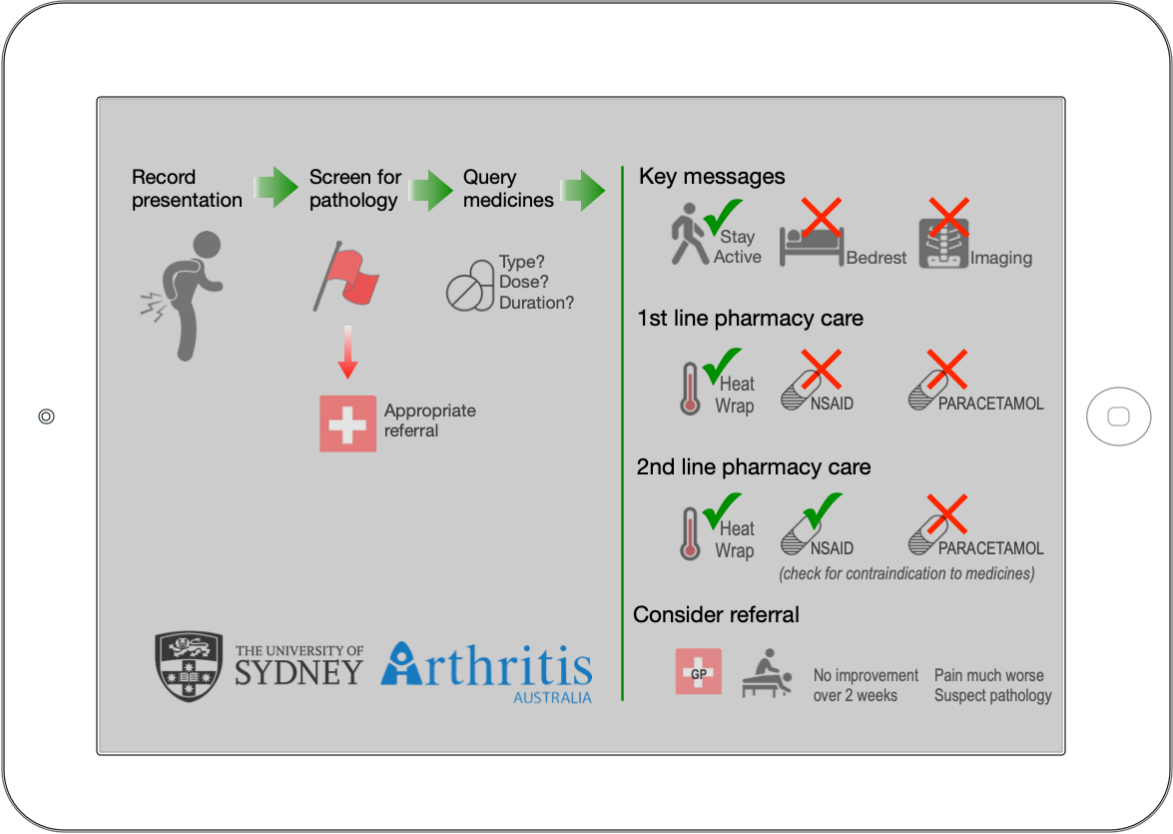
Clinical decision support system landing page showing clinical flow and scope of key messages. Tapping anywhere on this screen moves to the “clinical history” page.

The CDSS was designed in three components [22]: (1) knowledge base, (2) reasoning engine, and (3) interface (see Multimedia Appendix 1 for further explanation of design process). Briefly, the knowledge base included high level advice for the screening of serious pathology and early management of LBP [3,40-49]. The reasoning engine was coded from the knowledge base then refined using experts in LBP and community pharmacy to consider age, sex, results of screening questions, pain history, and up to three current medicines for LBP (Multimedia Appendix 2). Recommendations for the pharmacist are separated into key messaging for the pharmacy client, suggested medicine use, and referral options. The pharmacist progresses from a landing page (Figure 2), through to history, screening, and advice pages (Multimedia Appendix 3). Data input is via a touch interface (checkboxes, drop-down menus, and free-text input). The pharmacist can accept, modify, or reject advice generated by the CDSS. Finally, a custom letter for the pharmacy client is generated based on the pharmacist’s final recommendation (Multimedia Appendix 4).

The multidisciplinary team of experts were engaged at each stage of design, development, and internal testing. Decision trees were iteratively modified before coding decision logic and programming of the interface (Ionic Framework), then refined through multiple (>10) test cycles. Once the logic and interface were complete, each of the 408 unique decision combinations were checked for accuracy using the Web interface. Similarly, each of the clinical case vignettes (and CDSS-generated client handouts) were tested by the research team for language and accuracy before the interview phase.

### Usability Testing

After the completion of the internal testing, the next phase of usability was based on the recommendations of Yen and Bakken [34], where the community pharmacist interacted with the CDSS through a series of 5 case vignettes (system-user-task).

#### Participants

In all, 5 practising community pharmacists (with 5-27 years of clinical experience), not involved in the initial development phase, from 5 different community pharmacies in the Sydney metropolitan area were each presented with 5 case vignettes during a one-to-one interview. Inclusion criteria required pharmacists to have experience with computer use within pharmacy (eg, computer-based dispensing systems) and be comfortable with tablet computer use (eg, internet browsing). Previous studies suggest that with 5 participants, up to 80% of usability issues can be identified (including up to 100% of major usability issues) when a system is designed for a specific group of users [50,51].

#### Interview Procedure and Training

Each pharmacist was presented with the same 5 case vignettes role-played by the lead researcher (AD). Clinical scenarios included presentations of both nonserious and serious causes of LBP in adult and elderly populations. Cases 1 and 2 involved nonserious LBP, case 3 involved suspicion of an osteoporotic compression fracture, case 4 presented with nonserious low back and leg pain below the knee, and case 5 presented with LBP and a recent history of cancer (Multimedia Appendix 5).

The interviews were held in a location convenient to the pharmacist, usually in the designated clinical consultation space within the pharmacy. The pharmacist was required to interact with both the “client” (researcher) and the CDSS on an iPad Air (iOS 12.4, Apple Inc). Before beginning the case vignettes, the pharmacist was trained in the operation of the CDSS via interface “walk through.” Training also included a brief summary of the evidence underpinning the CDSS, explanation of the pharmacist-client interview process, and how to accept or reject the decision support offered by the CDSS.

The interview was conducted using a think-aloud protocol and employed an active intervention approach [52]. That is, the pharmacist was allowed to ask questions of the researcher during interaction with the “client.” Active intervention by the researcher was triggered when the pharmacist was unable to progress through the CDSS, sought clarification when interacting with the interface, or had questions at completion of the case (eg, reflecting upon management decisions generated by the CDSS). All instances of active intervention were logged and evaluated.

#### Data Collection

Four modes of data collection were used during the interview: (1) think-aloud protocols [53,54] with active intervention approach [52], (2) video/audio recording and screen capture during interaction with the iPad [55,56], (3) direct interview questions at the completion of interaction with the CDSS [26,57], and (4) completion of a survey instrument (system usability scale) [58,59]. The survey instrument was completed by the pharmacist at the end of the interview and without the researcher present (Multimedia Appendix 5).

### Evaluation

#### Evaluation of Usability Testing

Interviews were transcribed verbatim, then independently analyzed by two researchers with assistance by a third (AD, CS and AK) using a directed content analysis methodology, where key concepts from existing usability studies of health information technology methodology were used to inform initial coding categories [34]. Operational definitions for each category were determined based on the specific goals of the CDSS. Any redundant coding categories were collapsed. The analysis of pharmacist sentiment was categorized as “negative,” “neutral,” or “positive” in consultation with the research team [60]. Frequency of responses were tabulated first by category, then by sentiment (NVivo 12.5, QSR International). Interaction with the iPad was time-stamped to calculate duration spent on each page of the iPad, periods of pharmacist hesitation, and page navigation decisions. Responses to survey instruments were described, and a system usability scale was scored [61].

#### Level of Acceptance of Clinical Reasoning and Decision Support

At the completion of all case vignettes, each pharmacist was shown an overview of the clinical reasoning engine and then asked to reflect on the logic that informed the recommendation for each case. To quantify the level of acceptance for the core set of recommendations generated by the CDSS (self-care advice, medicines advice, and referral advice), the pharmacist’s acceptance (accept/not accept) was logged. Additional advice offered by the pharmacist relating to clinical management was entered in free-text fields on the iPad.

## Results

### Pharmacist Interview

All pharmacists completed 5 case vignettes on the Apple iPad. Pharmacists were exposed to cases in the same order. The total time taken to role-play all 5 case vignettes (excluding discussion on decision logic or system improvements) ranged from 14 min 35 seconds (Pharmacist #1) to 28 min 4 seconds (Pharmacist #2). Case vignettes that included nonserious LBP required less time (Cases 1 and 2: mean 3 min 40 seconds per case, SD 1 min 8 seconds) than cases that raised suspicion of serious causes of LBP (Cases 3-5: mean 4 min 46 seconds per case, SD 1 min 23 seconds).

### Evaluation of Usability Testing

#### Coding Categories

A total of 162 statements during the 25 interactions between pharmacists and “clients” were logged. Nine coding categories were identified using directed content analysis (*Ease-of-use, Consistency, Visibility, Navigation, Workflow, Content, Understandability, Clarity, and Acceptance)*. For final coding, the categories *Navigation* and *Workflow* were merged, and *Understandability* was defined under *Clarity,* which resulted in seven final categories. Statements were also coded by sentiment (negative, neutral, or positive). Table 1 describes each category, with statement frequency and representative examples. A total of 71 statements related to the CDSS interface, and 91 statements related to clinical information (content) provided by the CDSS.

**Table 1:** Coding categories with statement frequency and representative examples.

Coding category with subcategory		Sentiment frequency								Representative coded statements with sentiment	
		Negative^a^		Neutral^b^		Positive^c^		Total^d^			
**Interface**											
	Ease-of-use: commentary on the simplicity of operation of the CDSS^e^		16		3		7		26		Negative: (SCREENING page) “It would be a lot easier if it said, ‘I’ve just got some questions I want to ask you, and I just go through them regardless of what you told me.” (Pharmacist #5) Positive: “The App has simple language, it’s not complicated, not medical, so that it can be used by everyone. So that’s a good thing.” (Pharmacist #1)
	Consistency: commentary on the consistency of visual language or interaction model		2		7		4		13		Negative: “I’m pretty sure I did tick ‘history of malignancy’. I was surprised that when I ticked that it didn’t do what it did do with Betty.” (Pharmacist #5) Positive: (Reads letter) “OK, so it’s very similar to the others.” (Pharmacist #3)
	Visibility: commentary on the visibility of system capabilities and system status and navigational cues within the CDSS		7		6		3		16		Negative: (ADVICE page) “I didn’t notice this one. (points to medicine advice)” (Pharmacist #4) Positive: “The prompts are there, so it’s just something to get used to maneuvering... which isn’t very difficult because its laid out quite easily/quite nicely.” (Pharmacist #2)
	Navigation/workflow: observation and commentary on progression/sequence through the CDSS		2		12		2		16		Negative: “If we miss one of these pieces of information, does the App ask us to go back?” (Pharmacist #1) Positive: (HISTORY page) “I really liked this page. I think it’s easy to go through.” (Pharmacist #3)
**Clinical information**											
	Content: commentary on what information is/is not provided by the CDSS		6		9		12		27		Negative: (HISTORY page) “Maybe we could add another icon: ‘Pregnant or Breastfeeding’” (Pharmacist #4) Positive: (ADVICE page) “OK, so it actually knows it’s sub-therapeutic when I put sub-therapeutic input. That’s very good. That’s very good.” (Pharmacist #2)
	Clarity: commentary on the clarity of the information provided by the CDSS		1		9		5		15		Negative: (SCREENING page) “I know that it’s not an infection because they say, ‘I fell, and now I’ve got pain’, so it seems like I probably of shouldn't have asked the questions, but I still did because it was still there.” (Pharmacist #5) Positive: (MEDICINES page) “...to recommend Ibuprofen or Aspirin or whatever, then the dose that’s required. That’s really good. That’s really good.” (Pharmacist #1)
	Acceptance: commentary on the clinical value of the CDSS recommendations		6		9		34		49		Negative: (ADVICE page) “I’m not sure about ‘stay active’. I’m not sure it’s OK.” (Pharmacist #1) Negative: (ADVICE page) “..and she needs to see someone – like a specialist in this area to find out what is the reason – it is good to have an X-Ray.” (Pharmacist #4) Positive: (Reads letter) “OK. So, stay active. That’s really good.” (Pharmacist #2) Positive: (regarding use in practice) “I’d love it... I like the clinical part of my job. I was thinking of having something on pain management plans.” (Pharmacist #1)

^a^Negative: negative sentiment.

^b^Neutral: neutral sentiment.

^c^Positive: positive sentiment.

^d^Total: total sentiment count for subcategory.

^e^CDSS: clinical decision support system.

#### Pharmacists’ Statements Related to Interaction With the Interface

**Figure 3:**
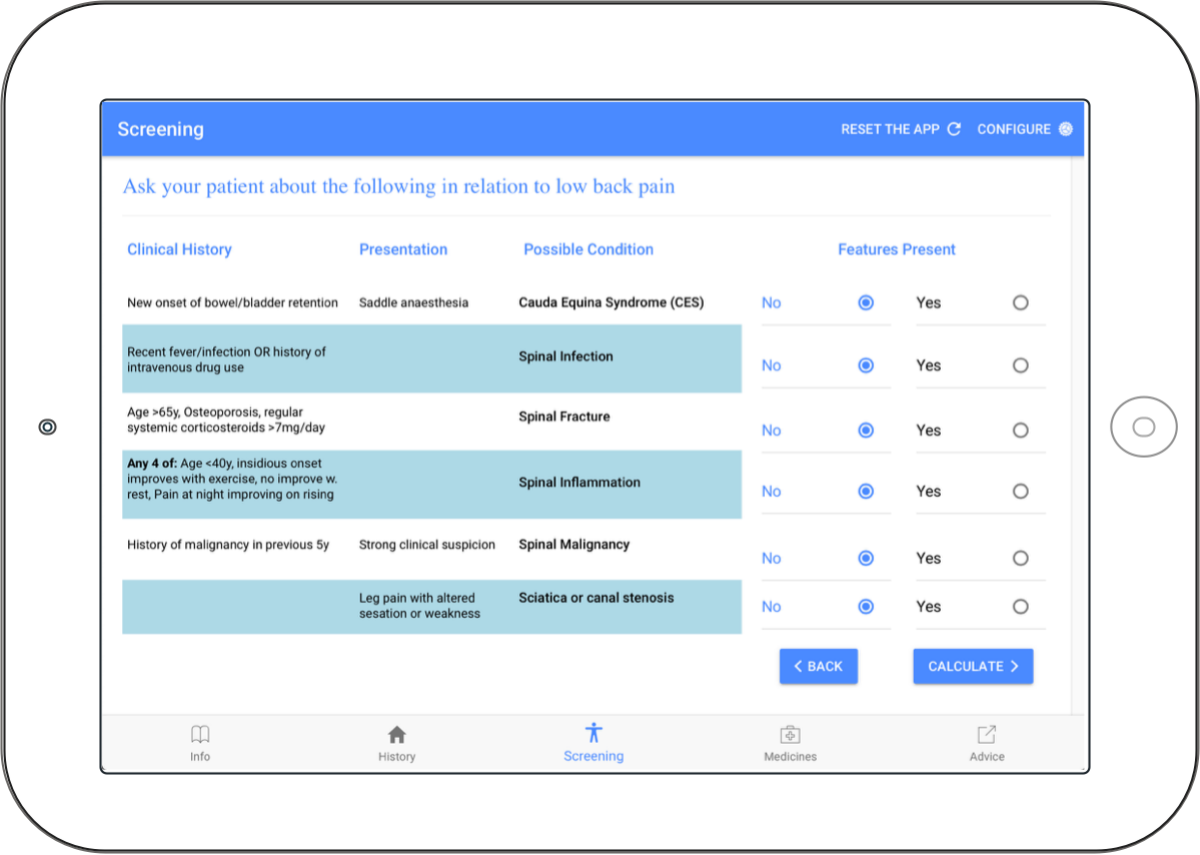
Clinical decision support system screening page for raising suspicion of a serious cause of low back pain. The “No” response is the default state.

The categories *Ease-of-use* and *Visibility* together accounted for 59% (42/71) of statements about the interface with a positive to negative comment ratio of 0.4 (7:16) and 0.4 (3:7), respectively. The majority of statements with negative sentiment involved interaction with the screening page (10/27 statements, Figure 3). The remainder scored with negative sentiment included comments on layout (eg, button position inconsistent) or visibility issues (eg, text size too small).

For example, statements with negative sentiment reported during interaction with the screening page included:

I find this part a bit long. I’m always reading through it (risk of spinal inflammation) … maybe it’s just me. Maybe I should just read it properly.

Pharmacist #2

[reads from iPad] Leg pain with altered sensation or weakness. So, I guess I didn’t see that part... is there a reason that that’s here (points to the 2nd column) – Oh, because it’s not a clinical history, yep.

Pharmacist #3

It’s just like you are trying to focus on the patient, so you are trying to do two things at once. If the patient was happy for me to pause, ‘cause you feel a bit awkward, just processing this whilst the patient is in front of you.

Pharmacist #4

I think that with this (SCREENING PAGE) is probably the hardest screen here because, like some of the questions I knew, like all of the cases so far, I know that it’s not an infection because they say I fell and now I’ve got pain, so it seems like I probably of shouldn’t have asked the questions.

Pharmacist #5

Statements with positive sentiment for the interface referenced the simplicity of layout, navigation, and language used (5/16 statements). In addition, statements with positive sentiment were made regarding integration with the pharmacist’s workflow (5/16 statements). Pharmacists’ statements relating to the operation of the CDSS (eg, “so I just press here,” and “then it comes out of the printer?”) comprised the majority scored with neutral sentiment (17/28 statements). Queries related to the operation of the CDSS decreased in frequency as each interview progressed.

#### Pharmacists’ Statements Related to Clinical Information

The categories *Content* and *Acceptance* together accounted for 84% (76/91) of statements related to clinical information provided by the CDSS, with a positive to negative comment ratio of 2.0 (12:6) and 5.7 (34:6), respectively. The remainder of statements related to *Clarity* of the clinical information provided by the CDSS. Statements with negative sentiment for *Content* (6/27 statements) included request for items absent from history (eg, pharmacists wanted to record current level of pain, whether pregnant or breastfeeding, and history of ulcer). Statements with negative sentiment for *Acceptance* (6/49 statements) included disagreement with, or questioning of, CDSS-generated advice in the categories self-care, medicines, and referral advice. For example:

Yes, but we always need to do further investigations to find out… and she needs to see someone – like a specialist in this area to find out what is the reason – it is good to have an X-Ray.

Pharmacist #4; case 1: nonserious cause of LBP

In my practice, a typical customer that you have just described will usually be on some kind of blood pressure medication – usually – which is why we always tend to recommend paracetamol first.

Pharmacist #5; case 2: recommendation to begin NSAID therapy

But, from the other point of view, would that narrow the amount of medicine that we recommend? So, from the business-Pharm point of view, would that exclude a lot of products?

Pharmacist #1; general comment at end of interview

Statements with positive sentiment for *Content* (12/27 statements) included what pharmacists considered to be the right information displayed at the right time. For example:

This is what's really interesting, is what really gives this one the meaning – I like the logic behind it.

Pharmacist #1; reacting to decision for suspicion of fracture

Yes, we do need to know this.

Pharmacist #4; points to increased risk of cancer

OK, so it actually knows it’s sub-therapeutic when I put sub-therapeutic input. That’s very good. That’s very good.

Pharmacist #5

Statements with positive sentiment for *Acceptance* (34/49 statements) included commentary on the clinical value of information generated by the CDSS in areas of self-care, medicines advice, and referral advice. These statements broadly reflected agreement with advice generated by the CDSS. For example:

Most of the time it was very logical, it was very rational and logical… It leads us to the right decision.

Pharmacist #1

Definitely! Its prompting you to ask questions. I must admit some of those questions we probably don’t always ask, but we need to be asking...

Pharmacist #2

OK, so just add on (stay active) Yep. OK. Instead of just seeing the GP straight away. OK. Cool!

Pharmacist #3; during the selection of self-care advice

We excluded some diseases which is good. I found out that she is not taking enough medicine.

Pharmacist #4

Yes, so I would say: using a heat wrap will also help, and I would say take the Voltaren 2-3 times per day with food – yes, it says this already!

Pharmacist #5

Similar to pharmacists’ questions relating to the interface, comments/questions related to clarification of CDSS-generated advice were scored with neutral sentiment. This type of question also decreased in frequency as each pharmacist moved through the 5 cases.

#### System Usability Scale

The system usability scale [59,61] was administered to each participant at the completion of the interview without the researcher present. Individual usability scores ranged from 82.5 out of 100 to 100 out of 100, which were interpreted as good to excellent usability, respectively [59]. The overall usability score was rated as excellent (mean score 92 out of 100, SD 6.5; 90th percentile compared with similar systems).

### Level of Acceptance of Clinical Reasoning and Decision Support

**Figure 4:**
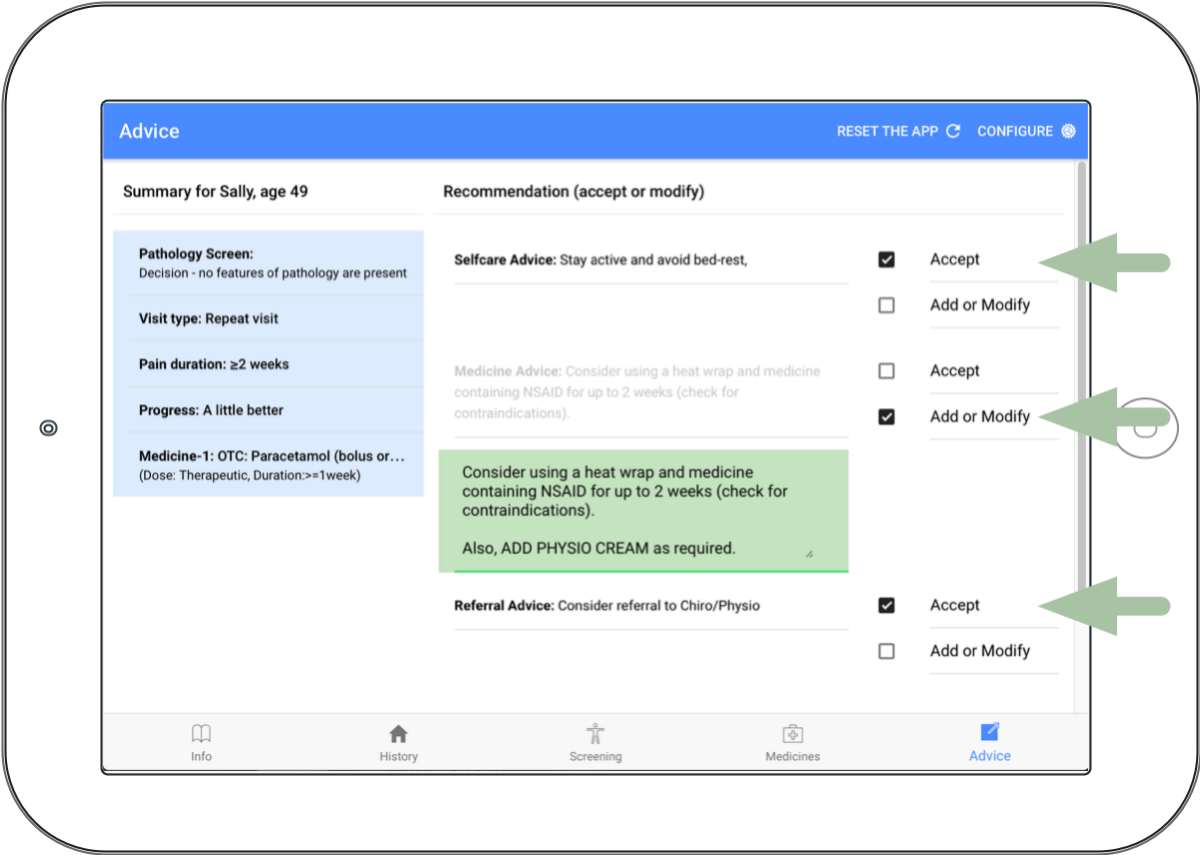
Advice page showing the pharmacist #1 acceptance of self-care advice, the augmentation of medicine advice by the pharmacist (“Also, ADD PHYSIO CREAM as required”), and the acceptance of referral advice (green arrows and green text box).

Across the 5 case vignettes, 70 recommendations were generated by the CDSS related to self-care advice, medicine advice, and referral advice. Pharmacists accepted 90% (18/20) of self-care recommendations, 100% (25/25) of medicines recommendations, and 88% (22/25) of referral recommendations. Of those accepted, pharmacists added to the advice for 8% (5/65) of the recommendations generated by the CDSS (eg, Figure 4). 

## Discussion

### Principal Findings

A CDSS was developed to enhance pharmacist care of LBP, underpinned by clinical practice guidelines and informed by a multidisciplinary team of experts that included consultation with community pharmacy. Community pharmacists rated the overall usability of the high-fidelity protype as good to excellent [59], despite expression of some negative sentiment in relation to guidance in screening for serious causes of LBP and interface inconsistency. There was a high level of acceptance for the advice generated by the CDSS for self-care, medicines, and referral, with pharmacists augmenting advice for a minority (5/65) of recommendations.

### Usability

Pharmacists reported a high level of usability based around simple use of language, logical workflow, brief consultation time, ability to customize advice, and convenience of a customized handout for the client. A number of usability issues were raised with regard to interface including page layout, text size, and button placement, which will be considered in the next phase of the CDSS refinement. The screening page (Figure 3) received the majority of negative comments and may reflect nonintuitive interaction with the layout of the screening page and/or lack of familiarity with the screening questions used to raise suspicion of serious causes of LBP. Although education for pharmacists in Australia contains topics on symptom recognition for differential diagnosis [62] and interprofessional referral [63], pharmacists expressed interest for more training on this topic, which is consistent with recommendations of Abdel Shaheed et al [64].

### Acceptance of the Clinical Support Provided by the Clinical Decision Support System

All pharmacists agreed that the information provided by the CDSS was applicable to the clinical scenarios presented and could potentially improve client-pharmacist encounters. One pharmacist disagreed with the messaging to avoid imaging and preferred to refer to medical care as a first option for nonserious LBP, but given the small sample, may not be representative of their peers. Pharmacists also commented that the CDSS helped them to ask more questions of the client with LBP and increased management options for LBP beyond their usual advice. However, it is unclear if the advice delivered by the CDSS in this setting would be superior to usual pharmacy care for LBP.

### Guidance for Pain Management in Community Pharmacy

Pharmacists commented that they appreciated guidance provided by the CDSS in relation to management, particularly for options beyond medicines advice. This aligns with recommendations of Abdel Shaheed et al [65] and others [11] on the potential benefit of tools/guidelines to support pharmacists when managing clients with LBP. Pharmacists also reflected on the current general lack of guidance to manage pain within pharmacy compared with the promotion and availability of management tools for other health conditions [5,7,8,14,66]. This view is consistent with results from a recent study by Abdel Shaheed et al [65] who found that pharmacists were receptive to implementing a disease state management program for LBP. One area highlighted by pharmacists was the lack of operational knowledge in relation to screening clients for serious causes of LBP, which has been highlighted previously [18]. Abdel Shaheed et al also found that pharmacists had both the willingness and capacity to increase knowledge in this area [32,64]. One goal of a training module integrated into the next version of the CDSS would be to empower the pharmacist with the skillset to raise suspicion of potentially serious underlying pathology, then inform clients of options for prompt medical review [43,45].

### Limitations

The small sample size may not be adequate to capture the full range of pharmacists’ views or usability issues thus limiting the generalizability of the results [67], particularly with regard to the level of acceptance. However, the sample size was appropriate for this stage of CDSS development [34,51]. That is, it was sufficient to identify major usability issues (eg, when screening for risk of serious disease), that the CDSS interface could be navigated with minimal training, and that decisions generated were logical and easy for the pharmacist to apply (in a simulated scenario). The method used to assess usability (think aloud with active intervention) may have enhanced task performance through researcher-induced bias [68] but allowed greater insight into the sections of the CDSS that required further development [52]. Another source of bias that may have enhanced task performance was the nonrandomized order of cases (case complexity was greater later in interview). This stage of CDSS development was to finalize design elements in the community pharmacy setting before testing with real clients [34]. In its current design, the CDSS does not integrate with existing electronic record systems in pharmacy, which would be necessary before advanced testing and would increase the chance of adoption by pharmacists [69]. One approach would be integration with existing disease state management systems [70], which was also suggested by pharmacists during testing.

### Comparison With Prior Work

This CDSS is the first tool that the authors are aware of to assist community pharmacists in first-line care for people with LBP. Other electronic decision support systems have been targeted at the primary care setting for the management of LBP [25] and chronic pain [26,27]. This CDSS differs from existing systems in that it aims to empower the pharmacist to offer evidence-based first-line care beyond medicines advice, and stepped referral options to allied health, primary, or emergency care based on presentation or symptom progression. The opportunity to enhance the pharmacist-client interaction, identified as lacking in other systems [27,71], has been built into this CDSS by allowing the pharmacist to modify management advice then provide a customized handout for the client.

### Future Direction

The next phase is to modify the CDSS with lessons learned from this usability study, then reevaluate during the next level of system development (integration with clients into the pharmacy setting) [34]. The CDSS will also be evaluated with respect to the credibility of advice and satisfaction with care from the perspective of clients with LBP. An education module on the evidence-based management of LBP could be delivered to pharmacists in conjunction with training for the CDSS, which would assist with knowledge of screening for pathology and give context to the guideline-based care options suggested by the CDSS. Future studies may establish if pharmacist training during the use of a CDSS within the clinical encounter improves both pharmacist and pharmacy client satisfaction with care.

### Conclusions

Despite many years of clinical guidelines for the management of LBP, significant evidence-to-practice gaps remain. This CDSS has been designed to provide a unique opportunity for community pharmacists to provide simple evidence-based advice for clients who present with LBP. Importantly the CDSS offers key messages of reassurance, to remain active, to use medicines appropriately, and to avoid inappropriate imaging.

## Appendix

Multimedia Appendix 1 Clinical decision support system design methodology.

Multimedia Appendix 2 Clinical decision support system decision tree.

Multimedia Appendix 3 Clinical decision support system data entry screens.

Multimedia Appendix 4 Client handout example generated by clinical decision support system.

Multimedia Appendix 5 Interview guide.

## Abbreviations

CDSS: clinical decision support system
LBP: low back pain
