# Supplementary material for: An Electronic Clinical Decision Support System for the Management of Low Back Pain in Community Pharmacy: Development and Mixed Methods Feasibility Study
Source: JMIR Med Inform. 2020 May 11;8(5):e17203. doi: 10.2196/17203 (PMC7248808; doi:10.2196/17203)
Supplement: Multimedia Appendix 1 [file medinform_v8i5e17203_app1.docx]

## Multimedia Appendix 1: CDSS design methodology

The CDSS was designed in three components: (i) knowledge base, (ii) reasoning engine, and (iii) interface [1].

### Knowledge base assembly

The knowledge base that underpinned the CDSS was developed in two sections: management of non-serious LBP specific to the community pharmacy setting; and use of red flags to screen for serious pathologies that require medical referral. The knowledge base for the management of non-serious LBP was informed by clinical practice guidelines [2-7]. Where clinical practice guidelines did not have specific recommendations relevant to the pharmacy setting, the knowledge base was augmented by systematic reviews of self-management strategies [8-10] and use of over the counter medicines [11-14]. Similarly, the knowledge base relating to the screening for serious pathology that can present as LBP was informed by clinical practice guidelines [3-7], and where necessary guidance on individual pathologies (red flags) including cauda equina syndrome [15], infection [16], fracture [17-20], inflammatory arthritides [21-23], malignancy [19,24], and radicular syndromes [8,25,26].

### Reasoning (inference) engine design

The reasoning algorithm considered the patient’s age, sex, red flag status and clinical history (episode duration, pain progression, repeat visit status, medicines usage history), to compute a core set of recommendations (key messages, medicines use, referral options). The logic for the reasoning algorithm was initially coded based on information from the knowledge base and literature on expectations of the client with LBP [27], then refined in consultation with pharmacists [28-30] and experts in the screening [18], diagnosis and management of low back pain [6,7,31,32]. Final recommendations were based on consensus (Multimedia Appendix 2).

The reasoning engine considered current use of up to three concomitant medicines (including dose regimen and duration of use) that community pharmacists typically recommend for clients with LBP, or prescribed by general practitioners. In addition, the CDSS generated a warning for the client when multiple medicines of the same class (therapeutic duplication) were reported, or when the pharmacist judged a current medicine dose to be excessive. Given that the community pharmacist will often know more about the client than can be entered into the CDSS, the management recommendations can be accepted outright or modified/rejected by the pharmacist as appropriate [1].

### Interface design

The CDSS interface was designed around the elements of the clinical encounter, with interface design based on work by Kawamoto et al. [33] who described key factors in the successful implementation of a CDSS including provision of contemporaneous (time and place) decision support, providing actionable recommendations, and computerizing the process.

After the landing page, the pharmacist navigates: (i) clinical history, (ii) screening for pathology (red flags), and (iii) medicines used for the current episode. A fourth page presents a summary of client data alongside the synthesis of recommendations for management of the client with LBP (Multimedia Appendix 3). The pharmacist is able to accept, add, modify or delete advice generated by the CDSS. The main interface elements are check-boxes, radio-buttons and drop-down menu items. The interface is dynamic (i.e. the page order is modified based on user input). Once the pharmacist has chosen management, a single page PDF is generated that contains individualized advice for the client using appropriate language (Multimedia Appendix 4).

References

1. Berner ES, La Lande TJ. Overview of clinical decision support systems. 3rd ed. Berner ES, editor. Switzerland: Springer Nature; 2016. 313 p. ISBN: 978-3-319-31913-1 (eBook).

2. Qaseem A, Wilt TJ, McLean RM, Forciea MA. Noninvasive treatments for acute, subacute, and chronic low back pain: A clinical practice guideline from the American College of Physicians. Ann Intern Med; 2017; 166(7):514-30. PMID-28192789.

3. Chou R, Deyo R, Friedly J, Skelly A, Hashimoto R, Weimer M, et al. Noninvasive treatments for low back pain. Rockville (MD): Agency for Healthcare Research and Quality (US); 2016 [cited 17/11/2019]; Available from: https://www.ncbi.nlm.nih.gov/books/NBK350276/?report=classic

4. Chou R, Deyo R, Friedly J, Skelly A, Hashimoto R, Weimer M, et al. Nonpharmacologic therapies for low back pain: A systematic review for an American College of Physicians clinical practice guideline. Ann Intern Med; 2017; 166(7):493-505. PMID-28192793.

5. Chou R, Deyo R, Friedly J, Skelly A, Weimer M, Fu R, et al. Systemic pharmacologic therapies for low back pain: A systematic review for an American College of Physicians clinical practice guideline. Ann Intern Med; 2017; 166(7):480-92. PMID-28192790.

6. Koes BW, van Tulder M, Lin C-WC, Macedo LG, McAuley J, Maher C. An updated overview of clinical guidelines for the management of non-specific low back pain in primary care. Eur Spine J; 2010; 19:2075-94. PMID-20602122.

7. Maher C, Underwood M, Buchbinder R. Non-specific low back pain. Lancet; 2017; 389(10070). PMID-27745712.

8. Dahm KT, Brurberg KG, Jamtvedt G, Hagen KB. Advice to rest in bed versus advice to stay active for acute low-back pain and sciatica. Cochrane Database Syst Rev; 2010; (6):CD007612. PMID-20556780.

9. French SD, Cameron M, Walker BF, Reggars JW, Esterman AJ. Superficial heat or cold for low back pain. Cochrane Database Syst Rev; 2006; (1):CD004750. PMID-16437495.

10. Furlan AD, Giraldo M, Baskwill A, Irvin E, Imamura M. Massage for low-back pain. Cochrane Database Syst Rev; 2015; (9):CD001929. PMID-26329399.

11. Abdel Shaheed C, Maher CG, Williams KA, McLachlan AJ. Interventions available over the counter and advice for acute low back pain: systematic review and meta-analysis. J Pain; 2014; 15(1):2-15. PMID-24373568.

12. Machado GC, Maher CG, Ferreira PH, Day RO, Pinheiro MB, Ferreira ML. Non-steroidal anti-inflammatory drugs for spinal pain: a systematic review and meta-analysis. Ann Rheum Dis; 2017. PMID-28153830.

13. Ward MM, Deodhar A, Akl EA, Lui A, Ermann J, Gensler LS, et al. American College of Rheumatology/Spondylitis Association of America/Spondyloarthritis Research and Treatment Network 2015 recommendations for the treatment of ankylosing spondylitis and nonradiographic axial spondyloarthritis. Arthritis Care Res (Hoboken); 2016; 68(2):151-66. PMID-26401907.

14. Saragiotto BT, Machado GC, Ferreira ML, Pinheiro MB, Abdel Shaheed C, Maher CG. Paracetamol for low back pain. Cochrane Database Syst Rev; 2016; (6):CD012230. PMID-27271789.

15. Singleton J, Edlow JA. Acute nontraumatic back pain: Risk stratification, Emergency Department management, and review of serious pathologies. Emerg Med Clin North Am; 2016; 34(4):743-57. PMID-612935198.

16. Tsiodras S, Falagas ME. Clinical assessment and medical treatment of spine infections. Clin Orthop Relat Res; 2006; 444:38-50. PMID-16523126.

17. Vestergaard P, Rejnmark L, Mosekilde L. Fracture risk associated with different types of oral corticosteroids and effect of termination of corticosteroids on the risk of fractures. Calcif Tissue Int; 2008; 82(4):249-57. PMID-18414920.

18. Williams CM, Henschke N, Maher CG, van Tulder MW, Koes BW, Macaskill P, et al. Red flags to screen for vertebral fracture in patients presenting with low-back pain. Cochrane Database Syst Rev; 2013:1-37. PMID-23440831.

19. Downie A, Williams CM, Henschke N, Hancock MJ, Ostelo RWJG, de Vet HCW, et al. Red flags to screen for malignancy and fracture in patients with low back pain: systematic review. BMJ; 2013; 347:f7095-f. PMID-24335669.

20. Grossman JM, Gordon R, Ranganath VK, Deal C, Caplan L, Chen W, et al. American College of Rheumatology 2010 recommendations for the prevention and treatment of glucocorticoid-induced osteoporosis. Arthritis Care Res (Hoboken); 2010; 62(11):1515-26. PMID-20662044.

21. Mathieson HR, Marzo-Ortega H. Axial spondyloarthritis: Diagnosis and management. Prescriber; 2015; 25(23-24):32-6. PMID-600712671.

22. Poddubnyy D, van Tubergen A, Landewe R, Sieper J, van der Heijde D. Development of an ASAS-endorsed recommendation for the early referral of patients with a suspicion of axial spondyloarthritis. Ann Rheum Dis; 2015; 74(8):1483-7. PMID-25990288.

23. Van Hoeven L, Vergouwe Y, De Buck P, Han K, Luime J, Hazes J, et al. Assessing the best referral strategy for axial spondyloarthritis; several referral strategies evaluated in primary care patients with chronic low back. Ann Rheum Dis; 2015; 74:758. PMID-72153053.

24. Henschke N, Maher CG, Ostelo RW, de Vet HCW, Macaskill P, Irwig L. Red flags to screen for malignancy in patients with low-back pain. Cochrane Database Syst Rev; 2013; (2). PMID-23450586.

25. Mathieson S, Kasch R, Maher CG, Pinto RZ, McLachlan AJ, Koes BW, et al. Combination drug therapy for the management of low back pain and sciatica: Systematic review and meta-analysis. J Pain; 2019; 20(1):1-15. PMID-30585164.

26. Deyo RA, Mirza SK. Clinical practice: Herniated lumbar intervertebral disk. N Engl J Med; 2016; 374(18):1763-72. PMID-27144851.

27. Verbeek J, Sengers M-J, Riemens L, Haafkens J. Patient expectations of treatment for back pain: A systematic review of qualitative and quantitative studies. Spine; 2004; 29(20):2309-18. PMID-15480147.

28. Abdel Shaheed C, McFarlane B, Maher CG, Williams KA, Bergin J, Matthews A, et al. Investigating the Primary Care Management of Low Back Pain: A Simulated Patient Study. J Pain; 2016; 17(1):27-35. PMID-26456675.

29. Abdel Shaheed C, Maher CG, Williams KA, Day R, McLachlan AJ. Efficacy, tolerability, and dose-dependent effects of opioid analgesics for low back pain: A systematic review and meta-analysis. JAMA internal medicine; 2016; 176(7):958-68. PMID-27213267.

30. Abdel Shaheed C, Maher CG, Mak W, Williams KA, McLachlan AJ. Knowledge and satisfaction of pharmacists attending an educational workshop on evidence-based management of low back pain. Aust J Prim Health; 2014; 21(2):126-31. PMID-24802263.

31. Hartvigsen J, Hancock MJ, Kongsted A, Louw Q, Ferreira ML, Genevay S, et al. What low back pain is and why we need to pay attention. Lancet; 2018; 391(10137):2356-67. PMID-29573870.

32. McRae M, Hancock MJ. Adults attending private physiotherapy practices seek diagnosis, pain relief, improved function, education and prevention: a survey. J Physiother; 2017; 63(4):250-6. PMID-28967562.

33. Kawamoto K, Houlihan CA, Balas EA, Lobach DF. Improving clinical practice using clinical decision support systems: a systematic review of trials to identify features critical to success. BMJ; 2005; 330(7494):765. PMID-15767266.
