# Supplementary figures and images for: An Electronic Clinical Decision Support System for the Management of Low Back Pain in Community Pharmacy: Development and Mixed Methods Feasibility Study
Source: JMIR Med Inform. 2020 May 11;8(5):e17203. doi: 10.2196/17203 (PMC7248808; doi:10.2196/17203)

(logic for decision support v3.1)

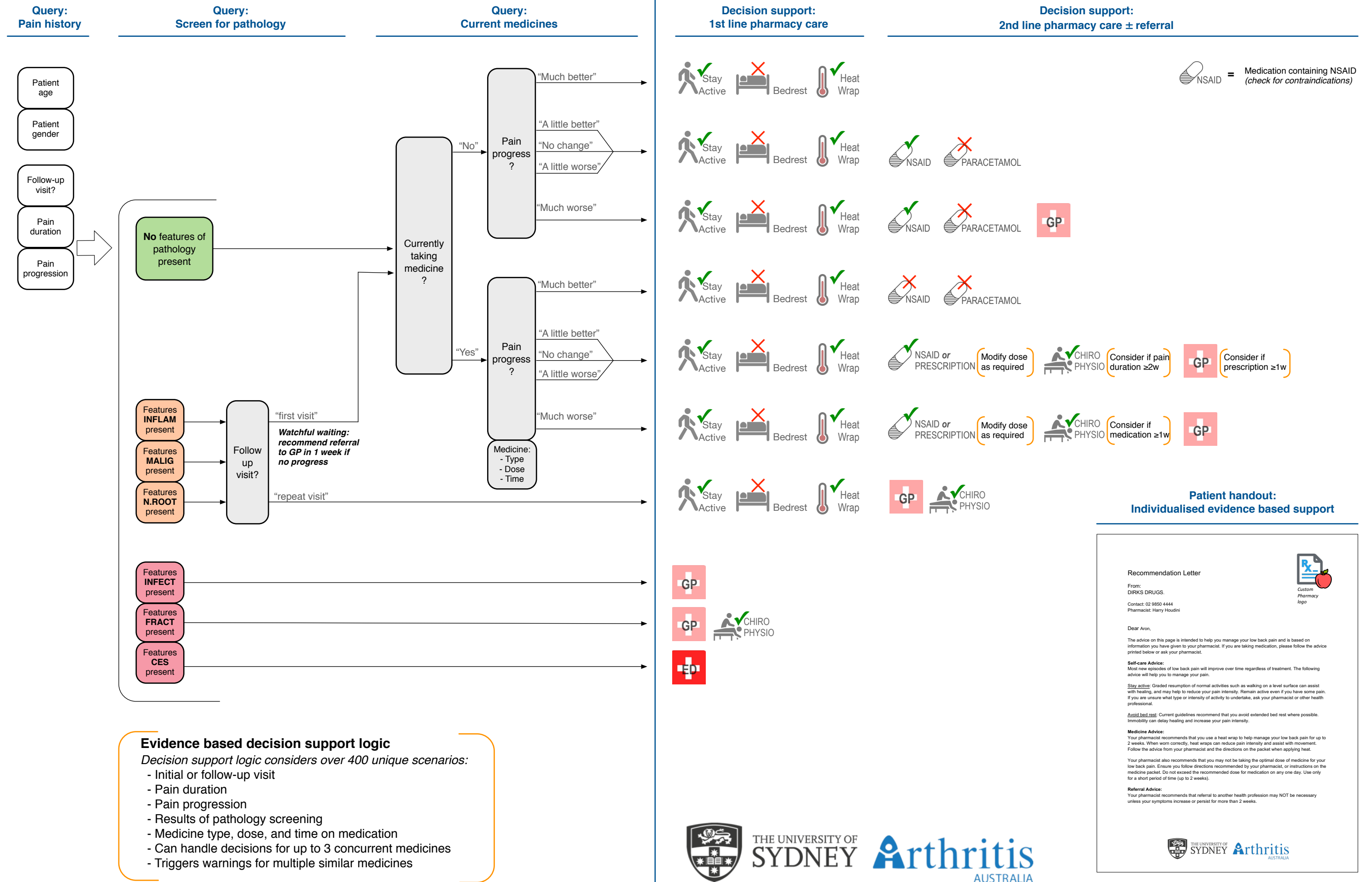

Supplement: Multimedia Appendix 2 [file medinform_v8i5e17203_app2.pdf]
