# Supplementary material for: An Electronic Clinical Decision Support System for the Management of Low Back Pain in Community Pharmacy: Development and Mixed Methods Feasibility Study
Source: JMIR Med Inform. 2020 May 11;8(5):e17203. doi: 10.2196/17203 (PMC7248808; doi:10.2196/17203)

Clinical decision support tool for low back pain in community pharmacy

Page 1. (after landing screen)

Pain history
- Name/age/gender
- New/followup
- Duration
- Progression

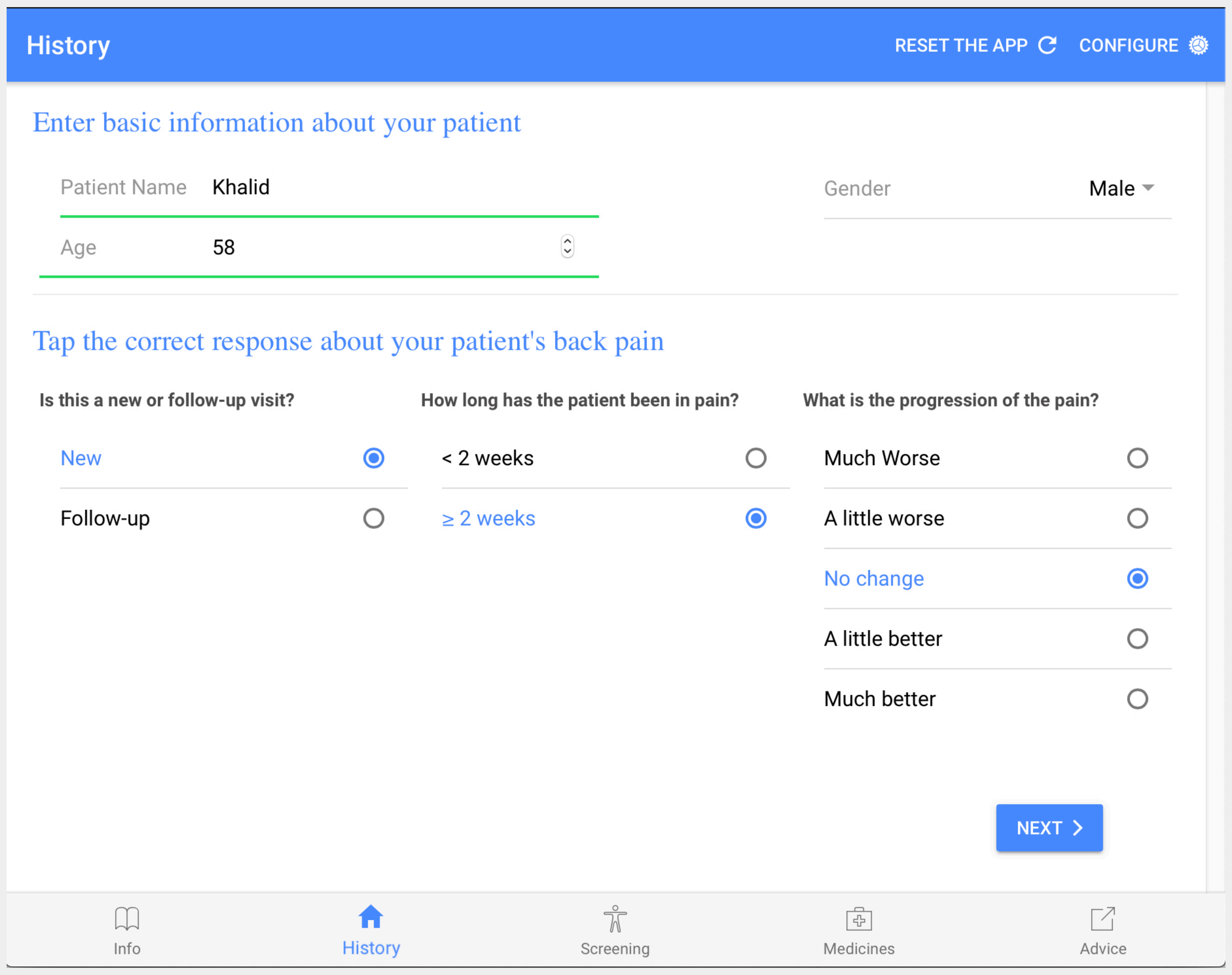

Page 2.

Current medicines
- Type
- Appropriate dose
- Time on medicine

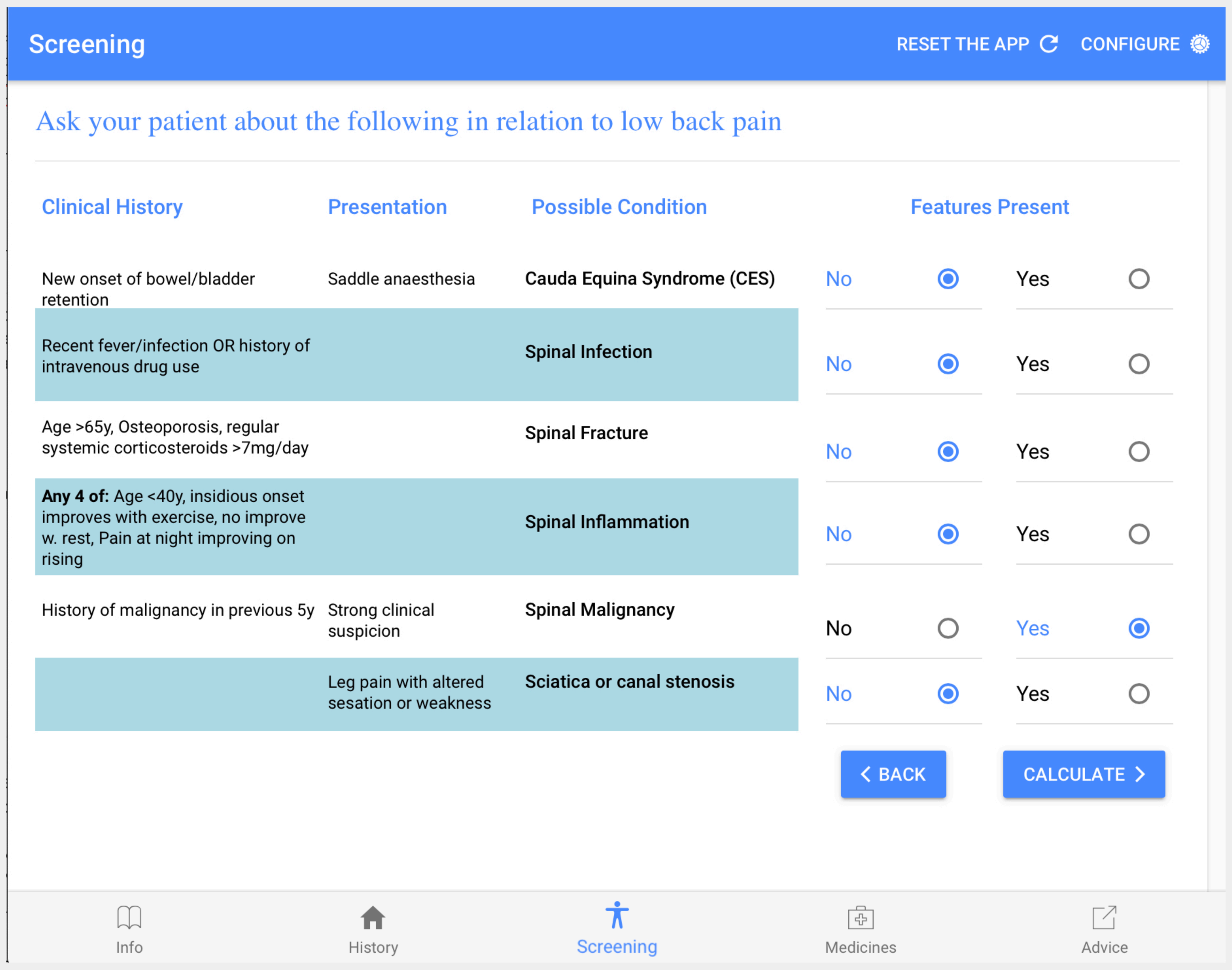

Page 3.

Current medicines
- Type (enter up to 3)
- Appropriate dose
- Time on medicine

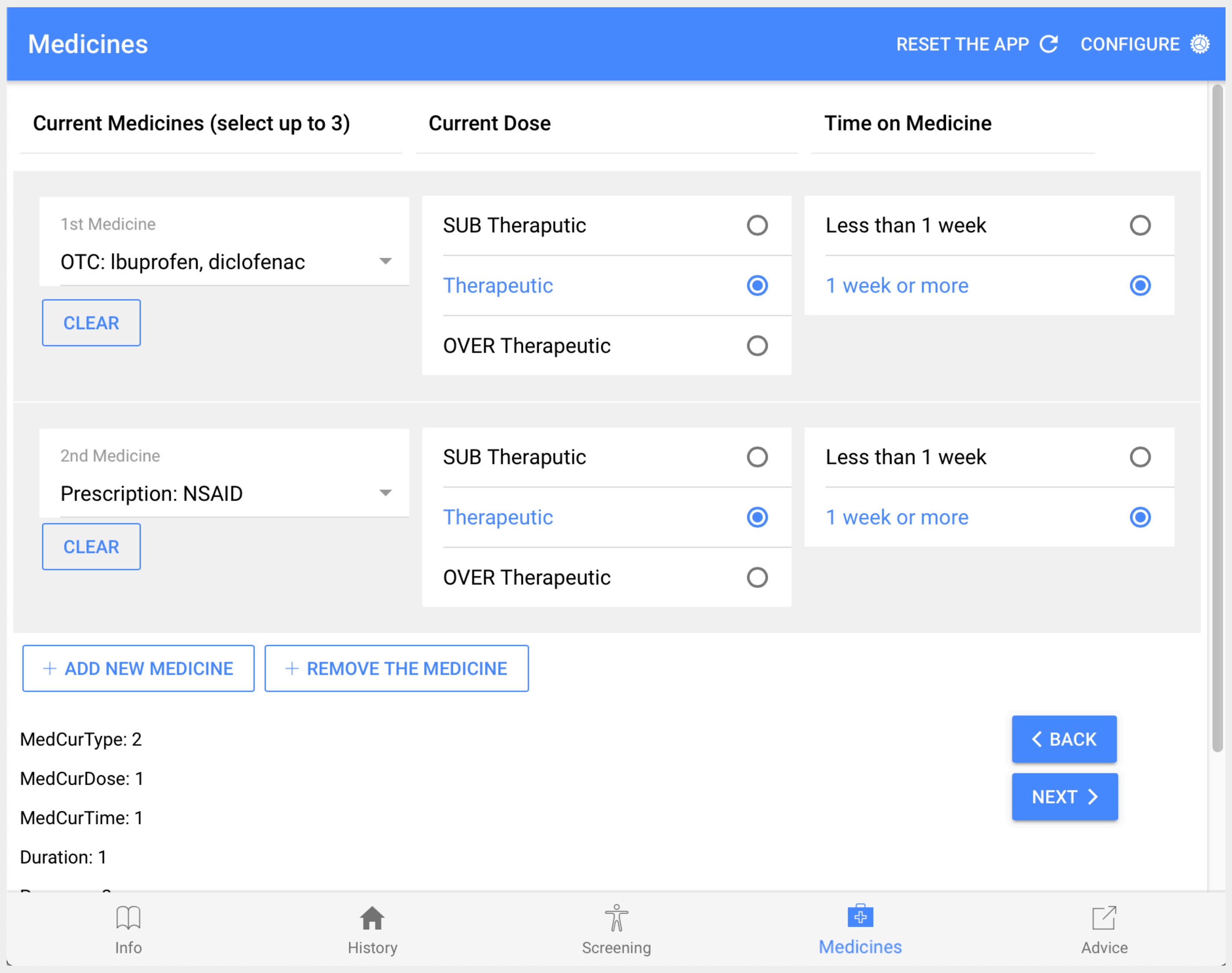

Page 4.

Decision support (± modify)
- Self-care advice
- Medicines advice
- Referral advice
- Custom advice

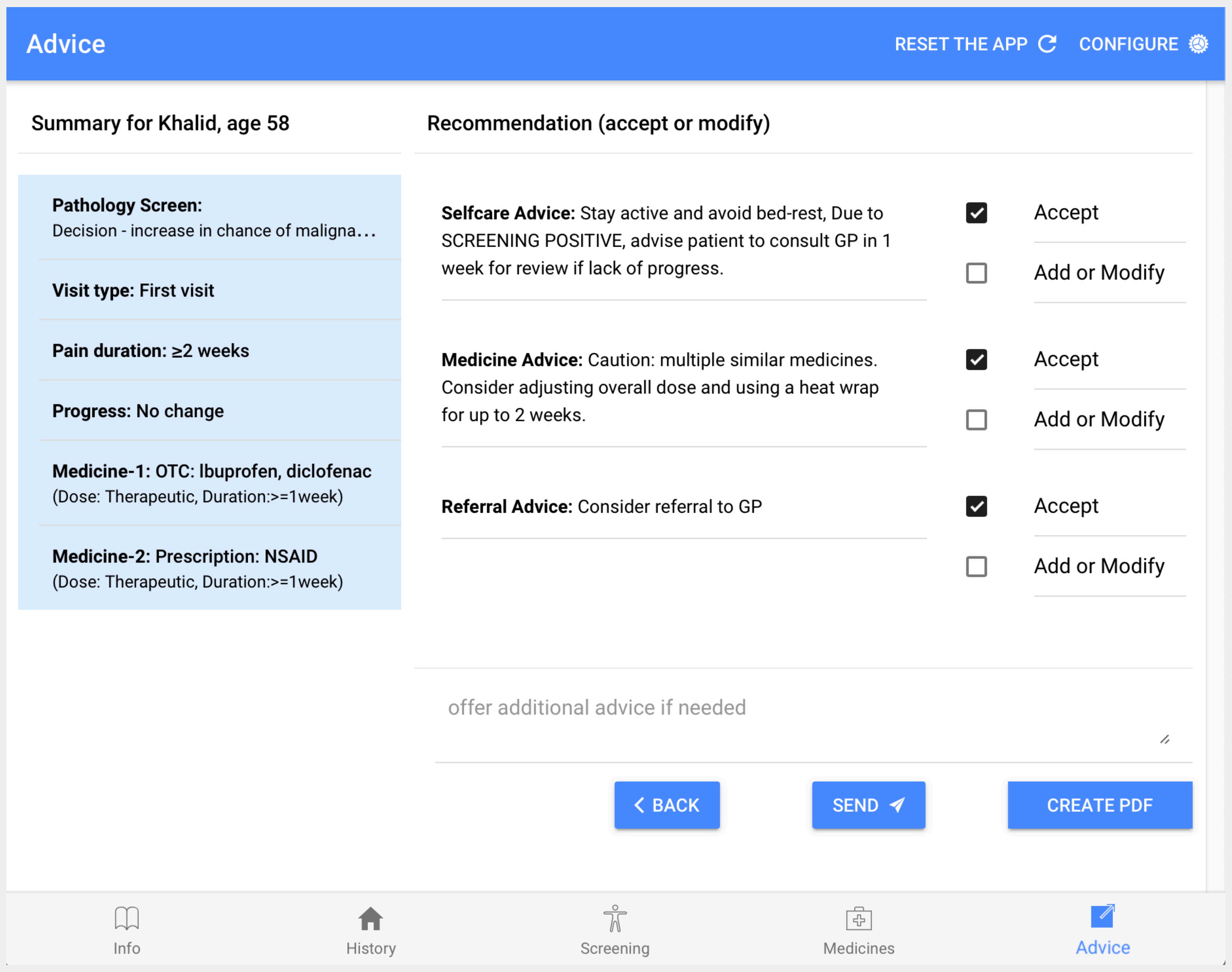

Supplement: Multimedia Appendix 3 [file medinform_v8i5e17203_app3.pdf]
