# Supplementary material for: An Electronic Clinical Decision Support System for the Management of Low Back Pain in Community Pharmacy: Development and Mixed Methods Feasibility Study
Source: JMIR Med Inform. 2020 May 11;8(5):e17203. doi: 10.2196/17203 (PMC7248808; doi:10.2196/17203)
Supplement: Multimedia Appendix 4 [file medinform_v8i5e17203_app4.pdf]

Pharmacy branding

Recommendation Letter

Contact: 00 1234 5678
Pharmacist: Joe Bloggs

Dear Sally,

The advice on this page is intended to help you manage your low back pain and is based on information you have given to your pharmacist. If you are taking medication, please follow the advice printed below or ask your pharmacist.

Self-care Advice:
Most new episodes of low back pain will improve over time regardless of treatment. The following advice will help you to manage your pain.

Stay active: Graded resumption of normal activities such as walking on a level surface can assist with healing and may help to reduce your pain intensity. Remain active even if you have some pain. If you are unsure what type or intensity of activity to undertake, ask your pharmacist or other health professional.

Avoid bed rest: Current guidelines recommend that you avoid extended bed rest where possible. Immobility can delay healing and increase your pain intensity.

Medicine Advice:
Your pharmacist recommends that you use a heat wrap to help manage your low back pain for up to 2 weeks. When worn correctly, heat wraps can reduce pain intensity and assist with movement. Follow the directions on the packet when using the heat wrap.

Your pharmacist also recommends that you may not be taking the optimal dose of medicine for your low back pain. Ensure you follow directions recommended by your pharmacist, or directions on the medicine packet.

- Do not exceed the recommended dose for this medication on any one day
- Do not use in conjunction with other anti-inflammatory tablets or gels (e.g. Aspirin, Ibuprofen, Diclofenac, gel-based medication)
- Always take the medication as directed with food
- Use only for a short period of time (up to 2 weeks)
- Excessive use can be harmful and increase the risk of heart attack, stroke or liver damage.

Referral Advice:
Your pharmacist recommends that referral to another health profession may NOT be necessary unless your symptoms increase or persist for more than 2 weeks.

THE UNIVERSITY OF SYDNEY Arthritis AUSTRALIA
