# Supplementary material for: An Electronic Clinical Decision Support System for the Management of Low Back Pain in Community Pharmacy: Development and Mixed Methods Feasibility Study
Source: JMIR Med Inform. 2020 May 11;8(5):e17203. doi: 10.2196/17203 (PMC7248808; doi:10.2196/17203)
Supplement: Multimedia Appendix 5 [file medinform_v8i5e17203_app5.pdf]

Aron Downie *PhD candidate*
Professor Chris Maher *Professor, Sydney School of Public Health*
A/Prof Mark Hancock

An electronic decision support tool to assist management of low back pain by community pharmacists – Interview script

Phase 1: tool validation interview script and discussion log

Introduction (~5 minutes)

The researcher welcomes the participant and finalizes consent for phase 1.

> *Good morning/afternoon, my name is Aron Downie. I am a researcher from University of Sydney Medical School. Thank you for agreeing to participate in this interview.*
>
> *Today I will walk you through up to 5 simulated case scenarios of a client who presents to you with low back pain. You will use custom iPad application to enter clinical information during the simulated case consultation. I will be presenting the cases to you.*
>
> *The interview will be in three parts:*
>
> i. *The iPad application walk-through to familiarise yourself with the tool*
> ii. *Discussion during case scenarios where you will interact with the application whilst 'thinking-aloud'. Feel free to comment on both the clinical case and your experience with the iPad interface. Any interface or case issues encountered that we did not discuss during the case scenarios can be discussed at the end.*
> iii. *You will be given a short survey to assess usability of the tool generated by the application.*

Musculoskeletal Health Sydney
School of Public Health
The University of Sydney
PO Box M179
Missenden Road NSW 2050 Australia

T +61 2 8627 6263
F +61 2 8627 6262
E aron.downie@sydney.edu.au
E christopher.maher@sydney.edu.au
sydney.edu.au

ABN 15 211 513 464
CRICOS 00026A

Step 1: App walkthrough (~5 minutes)

- Both the researcher and participant step through the app for purpose familiarisation. Questions relating to the workflow and interaction style can be discussed as needed.
- Prompt*: Practice talking aloud while completing the sample clinical scenario*

Step 2: Case scenarios and qualitative evaluation (~35 minutes)

- **Begin** Audio-visual recording [after checking consent to record has been granted]
- Up to 5 case scenarios (**Appendix 1**) are read aloud by the researcher.
- After each case presentation, the researcher will answer any questions, and will prompt the participant to *"keep on talking"*.
- Any interface or algorithm issues will be transcribed onto a paper form (**Appendix 2**) by the researcher while possible solutions for improving the interface are be discussed.
- **Stop** Audio-visual recording

Step 4: Quantitative evaluation (~10 minutes)

- Hand the participant the printed survey instrument OR complete same instrument online (**Appendix 3**)

**Participant Details**	
Name:	*Interview start time:*
Date:	*Interview end time:*
Video Recoding Y/N	
Audio Recording Y/N	

THE UNIVERSITY OF SYDNEY

Appendix 1: Simulated case scenarios

1) Sally, a 49yo female, presents c/o low back pain duration about 1 week, after tripping up a flight of stairs. Her pain is not improving.

   a) Sally denies any leg pain but feels a little weak has no change to bowel/bladder function; denies fever; no recent history of cancer. Sally appears well otherwise.
   b) Presently no exercise due to LBP.
   c) Sally takes 1 paracetamol (500mg) twice daily (bd) for pain for last few days
   d) Suggested diagnosis: **nonspecific low back pain**
   e) Suggested management:
      - Stay active
      - Avoid prolonged bed rest
      - Use a heat wrap for up to 2 weeks
      - Increase paracetamol dose to therapeutic level and use for up to 2 weeks
      - No referral necessary today

Re-presentation to pharmacy – 2 weeks later

2) Sally, a 49yo female, presents again c/o low back pain duration about 3 weeks, after tripping up a flight of stairs. Her pain is a little better but has not gone away.

   a) Sally denies any leg pain but feels a little weak has no change to bowel/bladder function; denies fever; no recent history of cancer. Sally appears well otherwise.
   b) Presently some exercise
   c) Sally takes 2 paracetamol (500mg) 3x daily (tid) for pain for 2 weeks
   d) Suggested diagnosis: **nonspecific low back pain**
   e) Suggested management:
      - Stay active
      - Avoid prolonged bed rest
      - Use a heat wrap for up to 2 weeks
      - Consider NSAID for up to 2 weeks
      - Consider referral to Chiro/Physio

3) Betty, a 69yo female, presents c/o low back pain and muscular spasm duration 3 days, after a fall in the garden. Her pain level is high and is getting worse.

   a) Betty denies any leg pain but feels generally weak; has no change to bowel/bladder; denies fever; no recent history of cancer. Takes regular corticosteroid therapy to help her breathing (5mg/day). Betty appears a little distressed.
   b) Paracetamol (2 tablets bd (1000mg)) gives minimal relief.
   c) Suggested diagnosis: **Consider vertebral compression fracture**
   d) Suggested management:
      - Do not advise OTC medicines
      - Recommend GP / allied health referral

4) Scott, an 18yo male, presents c/o low back pain duration 4 days, after lifting heavy object. His pain level is high and not decreased. He stays on the lounge due to his pain.

   a) Scott has pain from buttock into ankle; has no change to bowel/bladder; denies fever; no recent history of cancer. No regular medications
   b) Nurofen (2 tablets (400mg) 1x daily) gives minimal relief
   c) Suggested diagnosis: **Consider sciatica**
   d) Suggested management:
      - Watchful waiting for 1 week, then refer to GP/allied health if no improvement
      - Stay active
      - Avoid prolonged bed rest
      - Use a heat wrap for up to 2 weeks
      - Consider medication escalation (NSAID) to maximum therapy dose
        - Increase to 2 tablets (400mg) 3 x daily (tid)).

5) Khalid, 58yo male, presents c/o low back pain duration about 4 weeks. His pain level is not really reducing.
   a) Khalid has no leg pain; has no change in bowel/bladder; had diagnosis prostate cancer 4 years ago but is well otherwise.
   b) Medicine: Nurofen 2 tablets (400mg) 2x daily for 4 weeks
   c) Medicine: Prescription Voltaren 2 tablets (50mg) 2x daily for 1 week
   d) Suggested diagnosis: **nonspecific low back pain; consider cancer.**
   e) Suggested management:
      - Watchful waiting for 1 week, then refer to GP/allied health if no improvement
      - Multiple similar medicines. Consider adjusting overall dose (continue with prescription)
      - Stay active
      - Use a heat wrap for up to 2 weeks
      - Consider referral to GP

THE UNIVERSITY OF SYDNEY

Appendix 2: Interview discussion log – *researcher records responses below*

Suggested graphical user interface (GUI) improvements:
Suggestions to Improve Clinical Usefulness:
Comments about Integrating the Tool into Clinical Practice:

List problems encountered	Possible solutions

THE UNIVERSITY OF SYDNEY

Appendix 3: Survey instrument - Researcher offers participant the option to complete the survey in paper-based form (below) or online (using iPad).

(Researcher leaves the room)

System Usability Scale

Instructions: For each of the following statements, mark one box that best describes your reactions to your experience today.

	Strongly disagree				Strongly agree
1. I think that I would like to use this system frequently.	☐	☐	☐	☐	☐
2. I found this system unnecessarily complex.	☐	☐	☐	☐	☐
3. I thought this system was easy to use.	☐	☐	☐	☐	☐
4. I think that I would need the support of a technical person to be able to use this system.	☐	☐	☐	☐	☐
5. I found the various functions in this system were well integrated.	☐	☐	☐	☐	☐
6. I thought there was too much inconsistency in this system.	☐	☐	☐	☐	☐
7. I would imagine that most people would learn to use this system very quickly.	☐	☐	☐	☐	☐
8. I found this system very cumbersome to use.	☐	☐	☐	☐	☐
9. I felt very confident using this system.	☐	☐	☐	☐	☐
10. I needed to learn a lot of things before I could get going with this system.	☐	☐	☐	☐	☐
